# Transsynaptic transport of wheat germ agglutinin expressed in a subset of type II taste cells of transgenic mice

**DOI:** 10.1186/1471-2202-9-96

**Published:** 2008-10-02

**Authors:** Sami Damak, Bedrich Mosinger, Robert F Margolskee

**Affiliations:** 1 Department of Physiology and Biophysics, Mount Sinai School of Medicine, 1425 Madison Avenue, Box 1677, New York, New York, 10029, USA; 2 Department of Neuroscience, Mount Sinai School of Medicine, 1425 Madison Avenue, Box 1677, New York, New York, 10029, USA

## Abstract

**Background:**

Anatomical tracing of neural circuits originating from specific subsets of taste receptor cells may shed light on interactions between taste cells within the taste bud and taste cell-to nerve interactions. It is unclear for example, if activation of type II cells leads to direct activation of the gustatory nerves, or whether the information is relayed through type III cells. To determine how WGA produced in T1r3-expressing taste cells is transported into gustatory neurons, transgenic mice expressing WGA-IRES-GFP driven by the T1r3 promoter were generated.

**Results:**

Immunohistochemistry showed co-expression of WGA, GFP and endogenous T1r3 in the taste bud cells of transgenic mice: the only taste cells immunoreactive for WGA were the T1r3-expressing cells. The WGA antibody also stained intragemmal nerves. WGA, but not GFP immunoreactivity was found in the geniculate and petrosal ganglia of transgenic mice, indicating that WGA was transported across synapses. WGA immunoreactivity was also found in the trigeminal ganglion, suggesting that T1r3-expressing cells make synapses with trigeminal neurons. In the medulla, WGA was detected in the nucleus of the solitary tract but also in the nucleus ambiguus, the vestibular nucleus, the trigeminal nucleus and in the gigantocellular reticular nucleus. WGA was not detected in the parabrachial nucleus, or the gustatory cortex.

**Conclusion:**

These results show the usefulness of genetically encoded WGA as a tracer for the first and second order neurons that innervate a subset of taste cells, but not for higher order neurons, and demonstrate that the main route of output from type II taste cells is the gustatory neuron, not the type III cells.

## Background

In the past few years, there have been tremendous advances in our understanding of the mechanisms of taste signal transduction. Many components of the mammalian taste signal transduction cascade have been identified, including the sweet and umami responsive T1r receptors, the bitter responsive T2r receptors, α-gustducin, Gγ13, PDE1A, PLCβ2, Trpm5, and PKDs (for reviews see [1-3]). Despite these advances at the molecular level, the mechanisms of taste coding remain unclear: how does depolarization of a subset of taste receptor cells lead to discrimination between tastants of different quality? There has been an on-going debate for several decades as to how taste qualities are coded. One view, the across fiber pattern model, maintains that taste qualities are represented by patterns of activity across afferent nerve populations. In another view, the labeled-line model, taste qualities are encoded by particular subsets of taste receptor cells and the afferent neurons to which they synapse. The existence of both narrowly and broadly tuned gustatory neurons gives support to both theories [4-7]. The discovery that T1r1, T1r2 and T2rs do not co-express in the taste bud [8] suggests that subsets of cells expressing T1r1, T1r2, and T2rs may constitute the origin of a labeled line for umami, sweet and bitter, respectively. Furthermore, transgenic add-back of PLCβ2 driven by the T2r5 promoter to PLCβ2 knockout mice restores bitter but not sweet or umami tastes [9], and transgenic expression of a modified opioid receptor in T1r2-expressing taste receptor cells leads to preference for a synthetic opiate [10]. These data make a strong case for the existence of a labeled line in the periphery, but what happens beyond the taste cells in the gustatory neural circuits is still unclear.

The classical model in which a taste receptor cell responds to tastants with a depolarization event which leads to activation of the neuron it synapses upon has recently been challenged [11]. Several investigators have shown that most type II taste receptor cells, which express the signal transduction molecules for bitter, sweet and umami, do not express synaptic markers or voltage gated calcium channels, whereas most type III cells express synaptic markers but no signal transduction genes for sweet, bitter or umami [12-16] and that ATP, which is released from type II cells upon stimulation by tastants can activate type III cells [17,18]. Thus, it has been suggested that upon activation by a tastant, type II cells transmit the signal to type III cells, and that the type III cells activate the nerves [18]. However, another study showed that knocking out purinergic receptors expressed in the taste nerves but not in type III taste cells greatly reduces the nerve and behavioral responses of mice to sweet, bitter and umami compounds. These findings support the presence of direct communication between type II taste cells and the gustatory nerves [14,19,20].

Anatomic tracing of the neural circuits originating from "modality-specific" taste receptor cells may shed light on the functional organization of the taste circuitry in the taste bud, and in the peripheral and central nervous systems. Wheat germ agglutinin (WGA) has widely been used as a tracer of neural circuits because of its ability to cross synapses [21]. WGA and the related barley lectin have been expressed in transgenic mice to trace the olfactory, visual and gustatory neural circuits [22-25]. The work of Sugita and Shiba [24] showed that a WGA-DsRed expressed in subsets of taste receptor cells under the control of either the T2r5 promoter or the T1r3 promoter was transferred to the gustatory neural circuits and was detectable in the nucleus of the solitary tract, the pontine parabrachial nucleus, the thalamic gustatory area, and the gustatory cortex. Furthermore the WGA-DsRed marker localized to different areas of the gustatory nuclei, depending of whether it originated from T1r3 expressing cells or T2r5 expressing cells. However a very recent publication concluded that WGA expressed from the T1r3 promoter is undetectable beyond the second order neuron [26]. To independently trace the taste neural circuits and to get insight into the synaptic connections of T1r3-expressing cells, we produced transgenic mice expressing WGA under the control of the T1r3 promoter and investigated whether the transgene was transported to the neighboring taste cells, the geniculate, petrosal and trigeminal ganglia, and the medulla.

## Results

**Figure 1:**
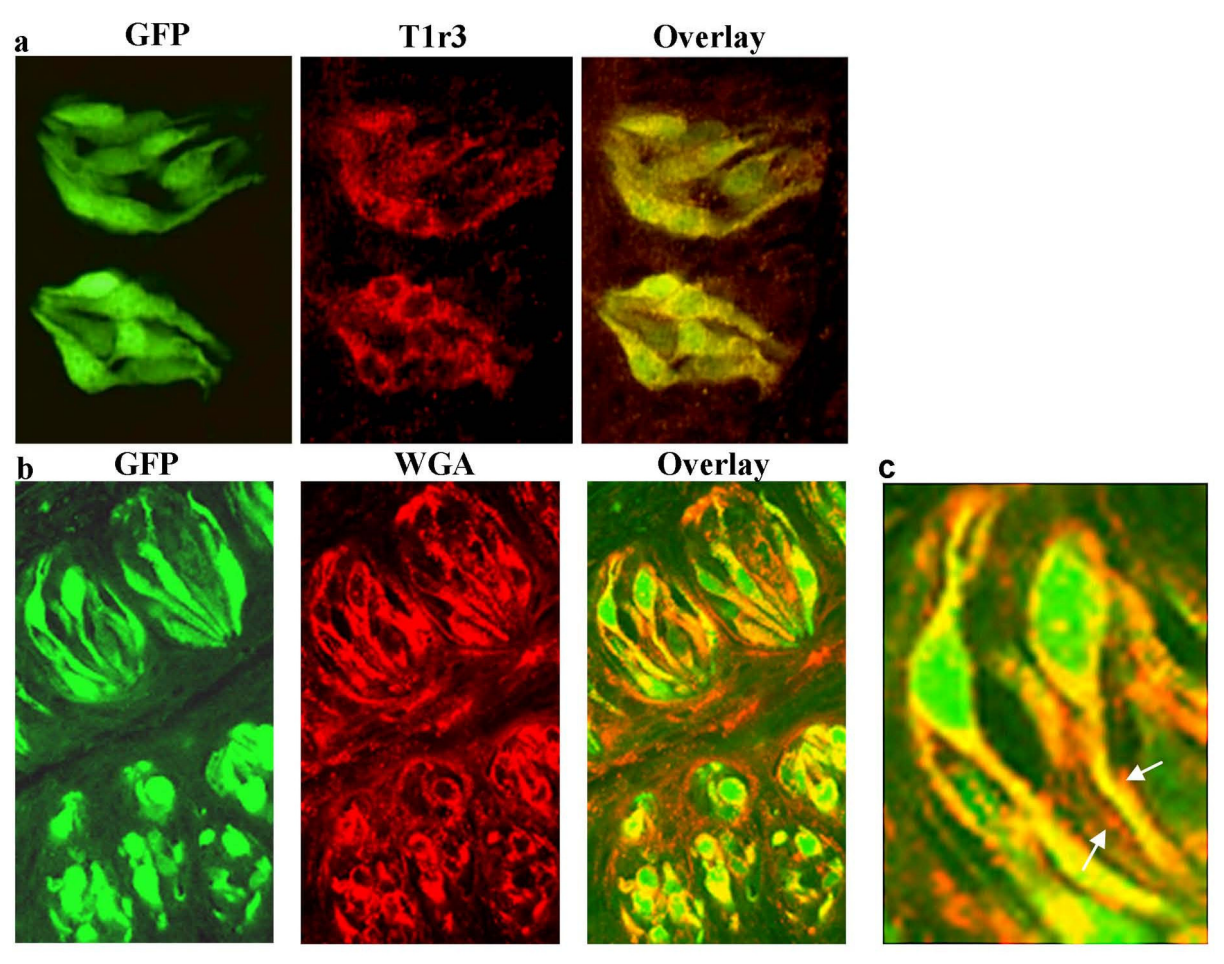
**Immunohistochemistry of a section from the circumvallate papilla of a T1r3-WGA-IRES-GFP transgenic mouse.  **a. Section immunostained with a T1r3 antibody (Cy3 fluorescence, red) and visualized for intrinsic fluorescence of the GFP transgene (green). Endogenous T1r3 and the GFP transgene are co-expressed in the same subset of taste cells. b. Confocal image of a section from the circumvallate papilla of a T1r3-WGA-IRES-GFP transgenic mouse, immunostained with a WGA antibody (Cy3 fluorescence, red) and visualized for intrinsic GFP fluorescence (green). WGA and GFP are co-expressed in the same subset of taste receptor cells. c. Higher magnification of rightmost panel of b., showing labeling of nerve fibers (arrows).

We produced transgenic mice expressing WGA-IRES-GFP under the control of the T1r3 promoter. IRES-GFP was included so that we can easily distinguish cells that produced WGA (because they also express GFP) from those to which WGA has migrated (which do not contain GFP). Immunohistochemistry with a T1r3 antibody showed co-expression of GFP and T1r3 in a subset of taste receptor cells, demonstrating that the WGA-IRES-GFP transgene was correctly expressed in the cells that normally express T1r3 (Figure 1a for co-expression in the CV, not shown for foliate and fungiform papillae). To determine if there was cell to cell transfer of WGA within taste buds, we carried out immunohistochemistry of CV sections with a WGA antibody, and examined the sections for WGA staining and GFP fluorescence. We found that WGA was co-expressed with GFP in a subset of taste cells, and was not expressed in the GFP non-expressing subset of taste cells. Nerve fibers within the taste bud also stained with WGA. These data demonstrate that the lectin migrates to the taste nerves, but not to neighboring cells in the taste bud (Figure 1b,c).

**Figure 2:**
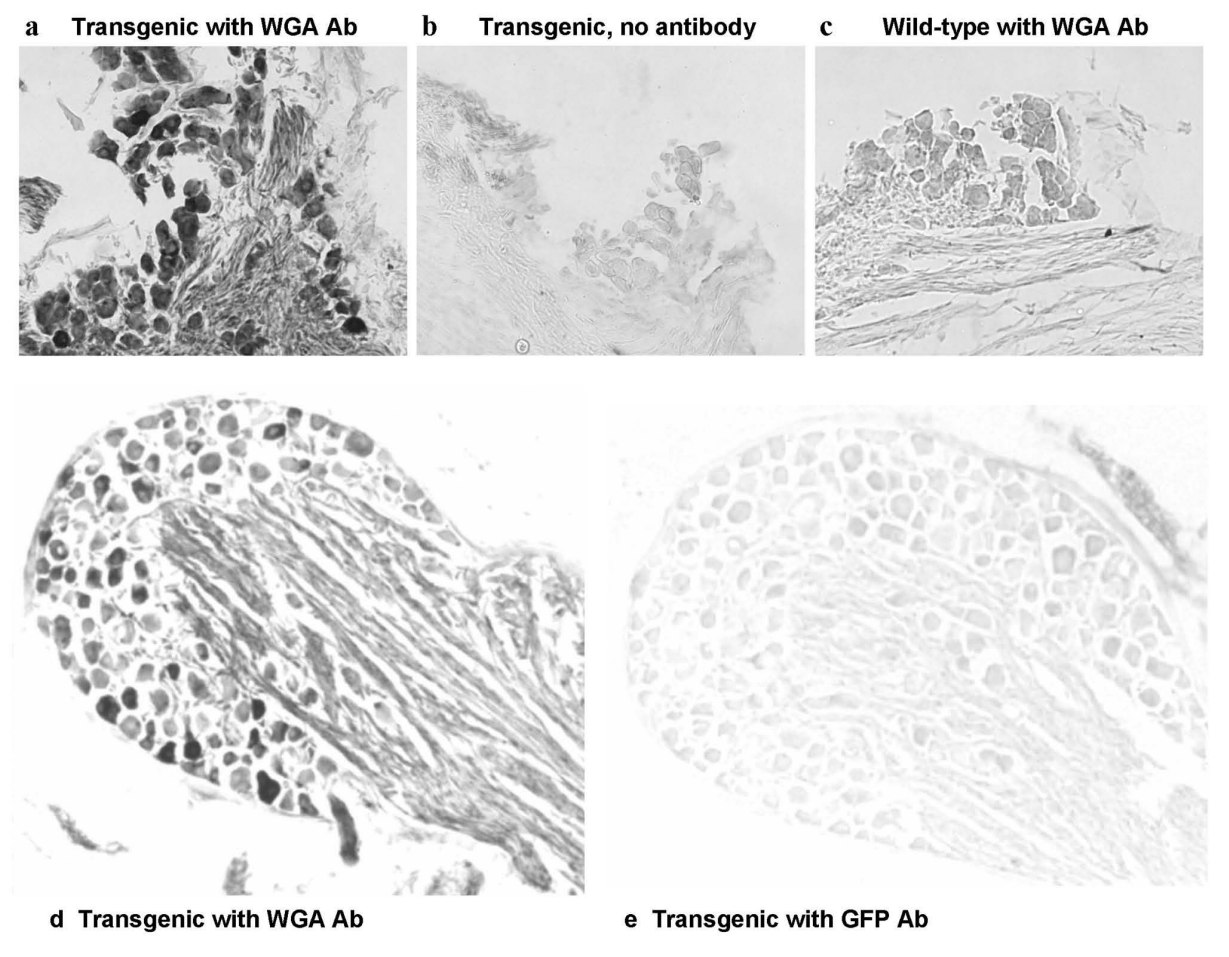
**Immunohistochemistry of sections from the geniculate (**a,b,c**) and petrosal (**d,e) **ganglia, showing staining with a WGA antibody in the ganglia from T1r3-WGA-IRES-GFP transgenic mice (**a,d**). **No staining was obtained when the primary antibody was omitted (b), or when a GFP antibody was used (e). No staining was obtained with the WGA antibody in the geniculate ganglion from a wild-type mouse (c).

To confirm that WGA was transported across the synapse to the neurons, we carried out immunohistochemistry on sections from the geniculate and petrosal ganglia of T1r3-WGA-IRES-GFP transgenic mice using WGA and GFP antibodies. These ganglia contain the cell bodies of the sensory neurons that innervate the taste receptor cells. Because of the intense autofluorescence of the ganglial cells, we used a non-fluorescent method for detection of WGA and GFP. We found WGA immunoreactivity in a large number of cells in the geniculate ganglion and in fewer cells in the petrosal ganglion, consistent with the geniculate ganglion containing mainly cell bodies of taste neurons and the petrosal ganglion containing cell bodies of both somatosensory and taste neurons (Figure 2). No WGA immunoreactivity was found in the ganglia of wild type littermates (Figure 2c). No GFP immunoreactivity was found in the ganglia of transgenic mice (Figure 2e), demonstrating that WGA was not produced in the ganglion, but migrated to the ganglion cells by crossing synapses.

**Figure 3:**
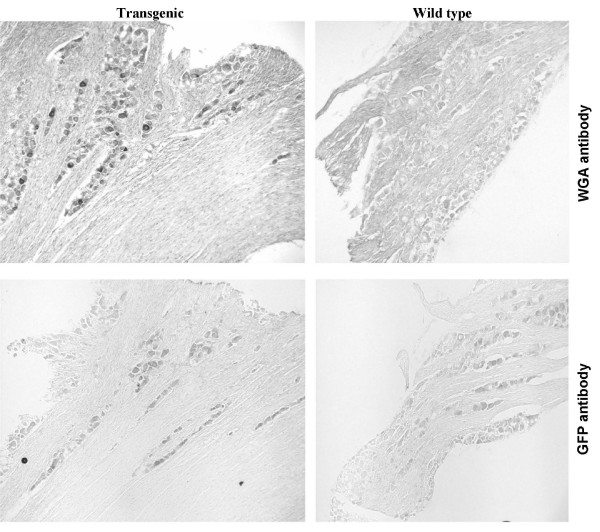
**Immunohistochemistry of sections from the trigeminal ganglia of T1r3-WGA-IRES-GFP transgenic (left) and wild-type (right) mice stained with antibodies against WGA (top) and GFP (bottom).** Immunoreactivity was found only in the ganglion of transgenic mice stained with for WGA.

We also immunostained sections from the trigeminal ganglia of T1r3-WGA-IRES-GFP transgenic mouse, and found surprisingly that a small number of cells from this ganglion also stained for WGA but not for GFP (Figure 3). These cells appear most abundant and their staining was strongest in the region of the ganglion that gives rise to the mandibular nerve. These data indicate that T1r3 or T1r3-containing cells may also be involved in initiating or modulating trigeminal responses (touch, pain, irritation). These results are consistent with the complexity of the response to food, integrating many senses including taste, olfaction and trigeminal response.

**Figure 4:**
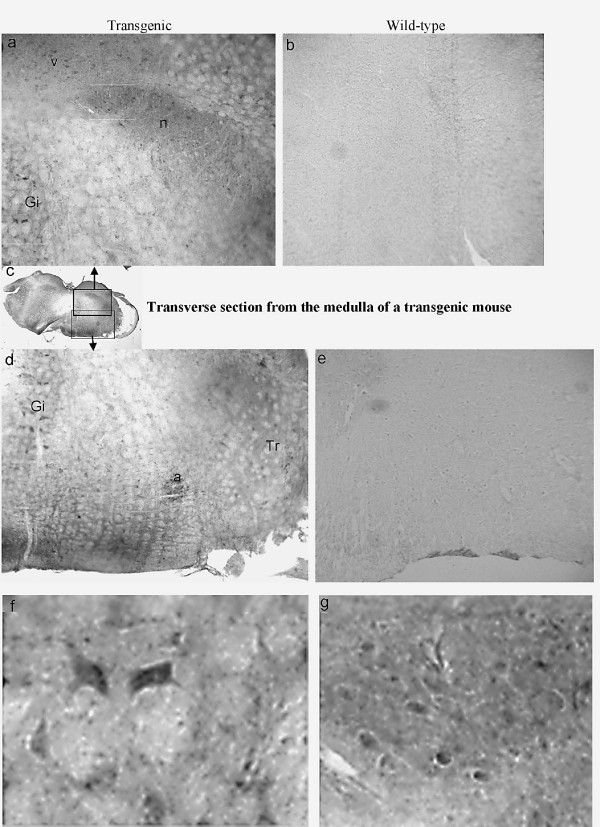
**Immunohistochemistry of a transverse section of the medulla of a T1r3-WGA-IRES-GFP transgenic mouse (Bregma -6.25) stained for WGA. **The entire section is shown in the insert in the middle of the figure (c), with black boxes showing the location of the high magnification pictures above (a) and below (d). Equivalent areas from a WT mouse are shown on the right (b,e). Higher magnification from the areas of panel c indicated by the white boxes are shown in panels f and g. Immunoreactivity for WGA is found in the NTS (n), the nucleus ambiguus (a), the vestibular nucleus (v), the trigeminal nucleus (Tr), and in the gigantocellular reticular nucleus (Gi) in the T1r3-WGA-IRES-GFP mouse but not in the WT mouse.

To determine if WGA was taken up by the second order neurons of the neural circuits that originate from the T1r3-expressing cells, we immunostained sections from the medulla of T1r3-WGA-IRES-GFP transgenic mice. As expected, WGA immunoreactivity was found in the nucleus of the solitary tract (NTS) but we also observed immunostaining in other areas of the medulla, including the nucleus ambiguus, the vestibular nucleus, the trigeminal nucleus and the gigantocellular reticular nucleus (Figure 4). The staining was weak but specific since no WGA immunoreactivity was observed in sections from equivalent areas from non-transgenic mice processed in parallel and with the same incubation and development times. Under high magnification, one can clearly see cell bodies stained above background, and occasionally staining of a neurite between two cell bodies (Figure 4f). No GFP immunoreactivity was found in any part of the medulla. We were not able to detect any WGA immunoreactivity in more central (tertiary) areas along the gustatory neural circuits, including the parabrachial nucleus, and the gustatory cortex. This is not surprising as the signal from WGA weakens every time it crosses a synapse, and the WGA signal was faint in the NTS. These experiments were repeated three times with always the same results.

## Discussion

The functional organization of taste cells in the taste buds and of the gustatory neural circuits are still subject to debate. WGA labeling of the neurons that synapse upon discrete subsets of taste receptor cells will help determine how the gustatory signal is transmitted within and beyond the taste buds.

We set out to determine if transgenic expression of WGA in a subset of mouse taste receptor cells could be used to this effect. Our data show that WGA originally expressed in T1r3-expressing type II taste receptor cells is transported to the geniculate and petrosal ganglia, and not to neighboring taste cells. WGA is believed to be taken up by and transported across synapses in vesicles. Together, these data suggest that the T1r3-expressing cells do make contact with chorda tympani and glossopharyngeal neurons. T1r3-expressing taste cells are type II cells which do not express the presynaptic molecules SNAP-25 and voltage-gated calcium channels required for synaptic transmission [16]. These cells activate the taste nerves by releasing ATP through pannexin hemichannels [19,20]. The recently reported observation of subsurface cisternae of smooth endoplasmic reticulum at regions where nerves and type II taste cells are in close apposition [14] suggests that this is the site of activation of the nerve by ATP released from the type II taste cell and may be the site where WGA crosses from taste cell to nerve.

As expected WGA immunostaining was observed in the geniculate and petrosal ganglia which contain the cell bodies of the nerve fibers of the chorda tympani and the glossopharyngeal nerves, respectively. Most neurons in the geniculate ganglion and many neurons in the petrosal ganglion were immunoreactive to the WGA antibody, although the intensity of the label varied from cell to cell. This pattern of staining is consistent with electrophysiological recordings from chorda tympani and glossopharyngeal single nerve fibers and geniculate ganglion cell bodies, which showed that there are broadly tuned and narrowly tuned fibers for each taste modality, and the fibers and cell bodies narrowly tuned to one modality also respond weakly to the other modalities [5,27,28]. We speculate that the strongly stained cell bodies may correspond to sucrose or MSG best neurons, the weakly stained ones may correspond to neurons narrowly tuned to other modalities and the cell bodies with intermediate staining may correspond to broadly tuned neurons. We cannot however exclude the possibility that WGA is released in the pericellular space in the taste buds and is nonspecifically taken up by nerves other that the ones in close contact with T1r3-expressing taste cells.

Surprisingly, WGA was also found in the trigeminal ganglion, which contains the cell bodies of neurons that relay somatosensory information from the head and neck to the brain. The most likely explanation is that WGA has originated from the recently discovered solitary chemoreceptor cells of the nasal epithelium [29], which also express T1r3 [26]. However it cannot be excluded that WGA has migrated from taste receptor cells to the trigeminal ganglion. It was initially believed, based on studies with fluorescent nerve tracers that trigeminal fibers surround the taste buds in fungiform papillae but do not enter them [30]. However, intragemmal nerve fibers immunoreactive for the vanilloid receptor TrpV1 were described, suggesting that trigeminal nerve fibers enter the taste buds [31]. Furthermore, after ontophoretic injection of a fluorescent dye in fungiform papillae, fluorescence was detected in some cells of the trigeminal ganglion [32]. This was attributed to direct injection of the perigemmal trigeminal fibers, but in the light of Ishida *et al *and our results it could well be transsynaptic transport from taste receptor cells. It is also possible that WGA was transported to the trigeminal ganglion from the brain, but we found no evidence of GFP expression in the medulla. Finally it is possible that WGA originated from other as yet undiscovered T1r3-expressing cells innervated by the trigeminal nerve.

We found a complex pattern of WGA localization in the medulla. In addition to the nucleus of the solitary tract, WGA immunoreactivity was found in the nucleus ambiguus, the vestibular nucleus, the trigeminal nucleus and in the gigantocellular reticular nucleus. This complex pattern is not surprising given the contribution of the taste sensation to several functions, e.g. swallowing, oromotor reflexes and conditioned taste aversion. The synapses crossed by WGA in the brain may be stimulatory or inhibitory and may not be sufficient alone to activate the neurons in which they occur. This may explain the difference between the pattern we found here and the more restricted pattern of fos-like immunoreactivity in response to sucrose [33] which results only from activated neurons. Others have found a more extensive labeling of the medulla by using a transsynaptic tracer. Bradley et al. [34] found that WGA conjugated to horse radish peroxidase injected in the circumvallate papilla of rats was transported to the solitary nucleus, the trigeminal system, the nucleus ambiguus, and the inferior salivatory nucleus. A similar pattern was reported by Hamilton and Norgren [35] after application of horse radish peroxidase to the cut lingual-tonsillar branch of the glosso-pharyngeal nerve. WGA transported through the trigeminal system also very likely contributes to the staining pattern we found in the medulla.

One limitation of this tracing approach is that the transport of WGA across synapses is both anterograde and retrograde, making it difficult to determine the order of the neurons in the neural circuits. The intensity of the WGA immunostain was strongest in the taste receptor cells and weakest in the medulla, and no GFP expression was detected in the ganglia or in the medulla. Therefore it is very likely that WGA migrated from taste receptor cells to the ganglia then to the medulla.

In this study, WGA is expressed from the promoter of a trangene, not knocked-in. Although unlikely, we cannot completely rule out ectopic expression of WGA in a small number of innervated cells outside the taste bud and migration of WGA to some nuclei in the medulla from those cells.

Two other publications have described WGA labeling of neural circuits originating from type II taste cells. The earlier publication by Sugita and Shiba [24] described neural circuits originating from T1r3 or T2r5 expressing cells and reported WGA staining in the third order taste neurons and in the gustatory cortex of transgenic mice expressing WGA-DsRed under the control of the T1r3 or T2r5 promoter. The main conclusion of Sugita and Shiba was that neural circuits originating from T1r3 or T2r5 expressing cells are mostly separated. In a very recent publication Ohmoto et al [26] described the neural circuits labelled by WGA expressed from the T1r3 promoter in transgenic mice. There are three major differences between the results of these two studies. First Sugita and Shiba detected WGA staining in the third order taste neurons and beyond, whereas Ohmoto et al did not. Second, Ohmoto et al detected WGA in the trigeminal ganglion whereas Sugita and Shiba did not. Third Sugita and Shiba reported that WGA was restricted to the taste areas in the central nervous system, whereas Ohmoto reported a wider pattern of WGA staining in the medulla. Our results are in full agreement with those of Ohmoto et al. and contrary to the main conclusions of Sugita and Shiba. Based on our results and those of Ohmoto et al., and contrary to Sugita and Shiba, the anatomical relationship between neural circuits originating from bitter responsive cells or sweet/umami responsive cells remains undetermined.

## Conclusion

Our results show the feasibility of using genetically encoded WGA as a tracer to label neural circuits that originate in a defined subset of taste receptor cells. It is especially useful for studies within the taste bud and of the first order gustatory neuron, and to some extent for the second order neurons. Beyond the NTS, however, this technique appears to be of minimal use without significantly enhancing sensitivity of detection. Using WGA tracing, we show that the main route of output from type II taste cells is the gustatory neuron and not other taste cells in the taste bud.

## Methods

### Transgenic mice

The WGA cDNA was a gift from Dr N.V. Raikhel. pIRES2-eGFP containing the encephalomyocarditis virus internal ribosome entry site (IRES) and enhanced green fluorescent protein (eGFP) was purchased from Clontech (Palo Alto, CA). The T1r3 sequence was subcloned from a BAC obtained by screening a C57BL6 mouse BAC library. The construct T1r3-13-WGA-IRES-GFP contains 5' to 3': 13 kb of mouse T1r3 including 5' flanking region and the entire 5' untranslated region, the WGA cDNA, IRES and eGFP. A stop codon was introduced in WGA at nucleotide 596 to remove a C-terminal peptide that prevents proper expression in mammalian cells [25]. The construct was separated from the vector by restriction digestion, purified from an agarose gel using a Qiagen kit and microinjected into B6C3 mouse zygotes according to standard methods [36]. Founders were bred to wild-type C57BL6/J mice and their transgenic offspring were used for further experiments.

### Immunohistochemistry

Mice were anesthetized with avertin and perfused with phosphate buffered saline (PBS) then with 4% paraformaldehyde in PBS. Tongue, brain and geniculate, petrosal and trigeminal ganglia were excised, fixed in 4% paraformaldehyde in PBS for 1 hour at 4°C, then transferred into 20% sucrose in PBS and stored at 4°C overnight. Fixed tongues were then embedded in Tissue-Tek OCT compound (Sakura, Tokyo, Japan) and 12–20 μm-thick cryostat sections were collected. Sections were treated for 30 minutes with 0.3% H_2_O_2 _to quench endogenous peroxidase activity and blocked in PBS containing 2% BSA, 1% horse serum, 0.3% Triton X-100 for 30 minutes at room temperature. For WGA and GFP staining, the ABC peroxidase kit (Vector Laboratories, Burlingame, CA) was used according to the manufacturer's instructions. Briefly, for WGA immunohistochemistry, a goat anti WGA antiserum raised against the entire protein (Vector Laboratories) diluted 1/200 was applied for 4 hours, followed by a biotin-conjugated horse anti goat IgG, then by a mix of avidin and biotin-conjugated peroxidase. The WGA antibody was cleaned with acetone powder of wild-type mouse brain before being used on brain sections. Diaminobenzidine tetrahydrochloride (DAB) was used for detection with a development time of 12 minutes. For GFP staining the same protocol was followed except that the primary antibody was biotinylated anti-GFP (1/300) raised against the entire protein (Vector Laboratories) and no secondary antibody was used. For T1r3 immunostaining, a rabbit primary and a Cy3-labeled goat anti-rabbit IgG secondary antibody were used as previously described [37]. The specificity of the antibodies against WGA and GFP is demonstrated by the absence of immunoreactivity in wild-type mice. The T1r3 antibody does not react with sections of taste buds from T1r3 KO mice [37]. For WGA or GFP detection, we used sections from transgenic animals and wild type littermates, processed in parallel under the exact same conditions and the same development time in DAB.

## Abbreviations

CV: circumvallate papilla; GFP: green fluorescent protein; IRES: internal ribosome entry site; NTS: nucleus of the solitary tract; WGA: wheat germ agglutinin;

## Authors' contributions

SD conceived the study, carried out most experimental work and wrote the manuscript. BM carried out the immunohistochemical study of co-expression of T1r3 and WGA, RFM contributed to the design of the study and revised the manuscript. All authors read and approved the final manuscript.
